# Adipokinetic Hormone Receptor Mediates Lipid Mobilization to Regulate Starvation Resistance in the Brown Planthopper, *Nilaparvata lugens*

**DOI:** 10.3389/fphys.2018.01730

**Published:** 2018-11-29

**Authors:** Kai Lu, Xinyu Zhang, Xia Chen, Yue Li, Wenru Li, Yibei Cheng, Jinming Zhou, Keke You, Qiang Zhou

**Affiliations:** ^1^State Key Laboratory of Biocontrol, School of Life Sciences, Sun Yat-sen University, Guangzhou, China; ^2^College of Life Sciences, Fujian Agriculture and Forestry University, Fuzhou, China

**Keywords:** adipokinetic hormone (AKH), adipokinetic hormone receptor (AKHR), lipid mobilization, starvation resistance, *Nilaparvata lugens*

## Abstract

Lipid storage must be efficiently mobilized to sustain the energy demands during processes of exercise or starvation. In insects, adipokinetic hormone (AKH) and brummer lipase are well-known regulators of lipid mobilization. We recently demonstrated that brummer-dependent lipolysis regulates starvation resistance in the brown planthopper, *Nilaparvata lugens*, one of the most destructive rice pests. The present work investigated the roles of the AKH signaling system in lipid mobilization during the starvation process in *N. lugens*. *Nl*AKHR is a typical G protein-coupled receptor (GPCR) and possesses high structure and sequence similarity to other insect AKHRs. Spatial and developmental expression profiles suggested that *Nl*AKH is released from the corpora cardiaca to activate *Nl*AKHR mainly expressed in the fat body. Starvation significantly induced the expression of *NlAKH* and *NlAKHR*, indicating a potential role of the AKH signaling system in starvation resistance. To reveal the functions of the AKH signaling system, a double-stranded RNA (dsRNA)-mediated knockdown of *NlAKHR* and *Nl*AKH peptide injection was performed. The results show *NlAKHR* silencing decreased the levels of 1,2-diacylglycerol (DAG) in the hemolymph and increased triacylglycerol (TAG) levels in the fat body, whereas *Nl*AKH injection led to a critical accumulation of DAG in the hemolymph and a severe reduction of TAG content in the fat body. Knockdown of *NlAKHR* resulted in prolonged lifespan and high levels of whole-body TAG, indicating an inability to mobilize TAG reserves during starvation. Conversely, the *Nl*AKH injection reduced the survival and accelerated TAG mobilization during starvation, which further confirms the role of *Nl*AKH in lipolysis. Moreover, *NlAKHR* silencing caused obesity in *N. lugens*, whereas *Nl*AKH injection depleted organismal TAG reserves *in vivo* and produced a slim phenotype. These results indicate that lipid mobilization is regulated by the AKH signaling system, which is essential for adjusting body lipid homeostasis and ensuring energy supplement during starvation in *N. lugens*.

## Introduction

The balance between lipid storage and mobilization is a critical characteristic of organismal energy homeostasis (Grönke et al., 2007). Most insects accumulate triacylglycerol (TAG), a strongly hydrophobic neutral lipid droplet with high energy content, as the primary lipid reserve for energy storage (Brown, 2001; Martin and Parton, 2006). TAG is mainly deposited in the fat body, an insect equivalent of adipose tissue, during periods of excessive food resources (Azeez et al., 2014). The ability to mobilize stored TAG reserves is important for the survival of insects under energy-demanding conditions. A tightly regulated balance between lipogenesis and lipolysis adjusts the TAG content in insects and matches acute energy needs in response to the fluctuation of environments (Grönke et al., 2007).

Two lipolytic systems, adipokinetic hormone (AKH)-mediated lipolysis and brummer lipase-dependent lipolysis, were reported to be involved in the regulation of TAG mobilization in insects (Stone et al., 1976; Grönke et al., 2005). This contrasts with vertebrates, where only one lipolytic system that involves adipocyte triglyceride lipase (ATGL), a homolog of insect brummer, is known to metabolize TAG (Zimmermann and Zechner, 2004). In insects, the levels of hemolymph lipid and carbohydrates are regulated by AKH, a peptide hormone which is thought to be functionally analogous to the mammalian glucagon (Lee and Park, 2004; Bharucha et al., 2008). AKH was first identified as an insect neurohormone that stimulates lipolysis and locomotor activity in *Locusta migratoria* (Mayer and Candy, 1969; Stone et al., 1976). To date, more than 60 different kinds of AKHs have been identified or predicted from genome sequencing projects as highly conserved peptide hormones with similar structural characteristics in insect species (Gäde and Marco, 2013). Functionally, insect AKHs have been shown to possess pleiotropic actions. For example, AKHs stimulate lipolysis of TAG into diacylglycerols (DAG) and the fat body-based conversion of glycogen into trehalose in response to starvation (Grönke et al., 2007). In addition to the energy-mobilizing activity, some other functions of AKHs have also been revealed, such as inducting foraging activity in starved *Drosophila melanogaster* (Lee and Park, 2004), stimulating midgut proteolytic activity in the flesh fly *Sarcophaga crassipalpis* (Bil et al., 2014) and playing an important role in oxidative stress (Bednářová et al., 2013). Recently, AKH signaling has also been shown to be strongly associated with insect reproduction (Lorenz, 2003; Lindemans et al., 2009; Attardo et al., 2012). AKH is synthesized and released from the corpora cardiaca (CC) into the hemolymph, then binds to a G protein-coupled receptor (GPCR) at the membrane of fat body cells, and eventually mobilizes lipid and carbohydrate reserves (Gäde and Auerswald, 2003; Caers et al., 2012; Gáliková et al., 2015).

The AKH receptor (AKHR) was first identified in the fruit fly *D. melanogaster* (Park et al., 2002) and the silkworm *Bombyx mori* (Staubli et al., 2002) as a rhodopsin-like GPCR with seven transmembrane-spanning alpha-helices, which is structurally and functionally analogous to the vertebrate gonadotropin-releasing hormone (GnRH) receptor (Lindemans et al., 2009). To date, AKHRs have been identified or predicted from genome sequencing projects in several other insect species (Supplementary Table 1). AKH signaling is achieved by binding peptide hormone with AKHR and then activating various cellular signaling pathways (Staubli et al., 2002). Insect AKH/AKHR signaling involves Ca^2+^ and cyclic adenosine monophosphate (cAMP) as intracellular messengers, and the signaling cascades of AKH/AKHR-stimulated lipolysis also involve the activation of protein kinase A (PKA) (Arrese et al., 1999; Van der Horst et al., 2001; Gäde and Auerswald, 2003). AKHR is ubiquitously expressed, however, it is highly accumulated in fat body, where AKH possibly functions *in vivo*. In the two-spotted cricket *Gryllus* *bimaculatus*, knockdown of *AKHR* led to the reduction of DAG and trehalose in the hemolymph whilst to elevated levels of TAG in the fat body (Konuma et al., 2012). Meanwhile, *AKHR* deficiency caused increased starvation resistance and decreased locomotory activity in the crickets (Konuma et al., 2012). Recently, it was shown that flies carrying *AKHR* mutations suffer from increased TAG and glycogen accumulation, supporting the idea that AKHR contributes to the lipolysis (Isabel et al., 2005; Bharucha et al., 2008). Along with these findings, AKHR mutant flies possess a phenotype of increased starvation resistance, which corresponds to the observation found in the AKH-deficient flies (Lee and Park, 2004; Grönke et al., 2007; Bharucha et al., 2008). Similar results were also obtained in *Rhodnius prolixus* (Alves-Bezerra et al., 2016) and *Bactrocera dorsalis* (Hou et al., 2017), wherein the silencing of *AKHR* reduced the levels of DAG and trehalose in the hemolymph and caused TAG accumulation in the fat body, which further underscores the central role of AKHR in insect lipolysis. The transcript expression of *AKHR* was also detected in the reproductive tissues, supporting the idea that energy metabolism mediated by the AKH signaling system might be regulated to meet the demands for female reproduction. In the tsetse fly *Glossina morsitans*, knockdown of *AKHR* resulted in an accumulation of stored lipids during pregnancy and caused a severe reduction of fecundity (Attardo et al., 2012). Recently, AKHR was reported to modulate female sexual traits, fecundity and flight duration in *B. dorsalis* (Hou et al., 2017). Therefore, lipolysis mediated by the AKH/AKHR signaling system is not only required for basic physiological functions, but also for survival during prolonged starvation and reproduction in insects.

The migratory brown planthopper *Nilaparvata lugens* is one of the most destructive rice pests in Asia, with a strong reproductive capacity. Moreover, many field populations have developed high levels of insecticide resistance. Since the resistance and fecundity both heavily rely on proper lipid metabolism, lipolytic systems related to lipid mobilization might significantly contribute to planthopper outbreaks (Lu et al., 2018; Zhou et al., 2018a). In previous studies, we identified brummer, a lipase which regulates lipid mobilization and starvation resistance in the planthopper (Zhou et al., 2018b). However, insect lipolysis is under control of multiple regulatory systems, and AKH-dependent mechanisms underlying the regulation of lipolysis have yet to be elucidated in this insect.

To reveal the role of AKH signaling pathway in lipid storage control, and to address the question of how these components orchestrate acute lipolysis in response to starvation, we identified the AKHR and analyzed its functions *in vivo*. We first investigated the evolutionary relationship of AKHR orthologs from other insect species, and then analyzed the expression patterns of *AKH* (Tanaka et al., 2014) and *AKHR* in different tissues and developmental stages. Then, the expressions of AKH/AKHR signaling system components under starvation conditions were characterized. Finally, the roles of this system in lipid mobilization and starvation resistance were tested by *AKHR* knockdown via RNA interference and AKH peptide injection. Our results suggest that the AKH/AKHR signaling system is indispensable for acute lipid mobilization and contributes to starvation resistance in the planthopper.

## Materials and Methods

### Insects and Sample Preparation

Individuals of *N. lugens* were maintained at Sun Yat-sen University, which were originally sourced from a colony from the South China Agriculture University in September 2008 (Lu et al., 2015). Insects were reared on fresh rice seedlings (Taichung Native 1) and kept at 26 ± 1°C with 65 ± 5% humidity under a 16/8 h (light/dark) photoperiod condition. Newly emerged females were collected and kept isolated until used for treatments.

Tissues, including head, midgut, ovary, fat body and epidermis, were dissected from 30 females that were 3 days old to investigate tissue-specific expression profiles. The females were anesthetized on ice and dissected in a precooled phosphate-buffered solution (PBS, 140 mM NaCl, 2.7 mM KCl, 10 mM Na_2_HPO_4_, and 1.8 mM KH_2_PO_4_; pH 7.4) under a stereomicroscope (SMZ745, Nikon, Tokyo, Japan). Dissected tissues were placed in a 1.5 mL RNase-free centrifuge tube and immediately frozen in liquid nitrogen and stored at -80°C until use.

Hemolymph was collected using a centrifugation method reported previously with moderate modification (Xu et al., 2006). Briefly, the females were anesthetized with carbon dioxide and the body wall of the thorax was pulled open using a tungsten needle. Then the punctured females were placed in a 0.5 mL RNase-free centrifuge tube with several small holes at the tube bottom. Next, the 0.5 mL tube was placed into a 1.5 mL RNase-free centrifuge tube and centrifuged at 9000 ×*g* at 4°C for 5 min. Thirty females were combined as one biological sample for hemolymph collection with three independent replicates.

### RNA Isolation, cDNA Synthesis and Cloning of *NlAKHR*

Total RNAs from whole bodies or different tissues were isolated using TRIzol reagent (Invitrogen, CA, United States) according to the manufacturer’s instructions. RNA integrity was confirmed by 1.5% agarose gel electrophoresis and RNA concentration was determined using a NanoDrop 2000C spectrophotometer (Thermo Fisher Scientific, West Palm Beach, FL, United States). In order to avoid genomic DNA contamination, total RNA was treated with RNase-free DNase I (Promega, Madison, WI, United States). A GoScript Reverse System (Promega) was used to synthesize the first-strand cDNA with 5 μg of total RNA in a 20 μL reaction mixture volume.

A partial cDNA sequence of putative *NlAKHR* was identified from a transcriptome of *N. lugens* (SRX023419) by performing a tBLASTn search using *D. melanogaster* AKHR sequence (NP_995639) as a query, and then verified by searching against a *N. lugens* genome database (PRJNA177647) (Xue et al., 2014). The full-length of *NlAKHR* was amplified using a SMART^TM^ RACE cDNA amplification kit (Clontech, Mountain View, CA, United States). Gene-specific primers were designed based on the cDNA partial sequence obtained as described above, and DNA polymerase GoTaq Master Mix (Promega) was used for RACE-PCR. Gene-specific outer primers and Universal Primer Mix (UPM) were used for the first round PCR (AKHR-F1 and UPM for 3′RACE; AKHR-R1 and UPM for 5′RACE) with the following amplification conditions: initial denaturation at 95°C for 2 min, followed by 35 cycles at 95°C for 30 s, 50°C for 30 s, 72°C for 2 min, and final extension at 72°C for 5 min. Nested PCR was carried out with gene-specific inner primers and Nested Universal Primer (NUP) (AKHR-F2 and NUP for 3′RACE; AKHR-R2 and NUP for 5′RACE) with 35 cycles of amplification (95°C for 30 s, 50°C for 30 s, 72°C for 1.5 min), and the diluted primary PCR amplification product was used as template. PCR products were separated by 1.5% agarose gel electrophoresis and purified (Tiangen, Beijing, China), subcloned into a pGEM-T easy vector (Promega) and transformed into *E. coli* DH5α competent cells (Tiangen). The inserted cDNA was sequenced by Life Technologies Company (Guangzhou, China).

### Sequence Characterization and Phylogenetic Analysis

The amino acid sequence of *Nl*AKHR was deduced from the corresponding cDNA sequence using the ExPASy Proteomics translation tool, and the transmembrane domains were predicted using the TMHMM server 2.0 (Krogh et al., 2001). The potential signal peptide position was predicted using the SignalP 4.1 Server (Mccarthy et al., 2004). Protein sequences for AKHR were downloaded from GenBank and aligned by ClustalW algorithm and a phylogenetic tree was constructed by the MEGA 6 software using the Maximum Likelihood (ML) method with a bootstrap of 1000 replicates (Tamura et al., 2013).

### Reverse Transcription PCR (RT-PCR) and Real-Time Quantitative PCR (qPCR)

RNA extraction and cDNA reverse transcription were performed as described above. Primers used for RT-PCR and qRT-PCR were designed by Primer 3 program (Untergasser et al., 2012) and are presented in Table 1. Partial cDNA fragments of *NlAKHR, NlAKH, NlTUB* (alpha 2-tubulin, FJ810204) and *NlRPS11* (ribosomal protein S11, FJ810197) were amplified using GoTaq Master Mix (Promega) under the following conditions: initial denaturation at 95°C for 2 min, followed by 30 cycles at 95°C for 30 s, 60°C for 30 s, 72°C for 45 s, and final extension at 72°C for 5 min. qRT-PCR was performed with a StepOnePlus^TM^ Real-Time PCR system (Applied Biosystems) using the UltraSYBR Mixture (CWBIO, Beijing, China) under the following reaction conditions: one cycle for 10 min at 95°C, followed by 40 cycles of 10 s at 95°C, 15 s at 60°C and 20 s at 72°C. A melting curve analysis was performed at the end of each qPCR to check the amplification specificity and to rule out the possibility of primer-dimer formation. All PCR reactions were carried out in triplicate, and at least two technical replicates were performed for each sample. The relative expression levels were calculated using the 2^-ΔΔCT^ method (Livak and Schmittgen, 2001) and the stable reference genes *NlTUB* and *NlRPS11* were used for normalization (Yuan et al., 2014).

**Table 1:** Primers used in this study.

Primers	Primer sequence
*For RACE*	
AKHR-F1	5′-GACTTCCCCATCGACATGCAGTTCAAC-3′
AKHR-F2	5′-ATGCTGCTGTCGGCTATAGGCAACTTC-3′
AKHR-R1	5′-CAGACCACGCTATCTCCAACGGCAT-3′
AKHR-R2	5′-AGGATGGAAAGGACGGTGAAGTTGC-3′
*For RT-PCR*	
AKH-F	5′-CACGGCGCAGGTCAACTTCT-3′
AKH-R	5′-TTCGCATGAAGTGCAGTTGT-3′
AKHR-F	5′-CAAAGAACCCCAGCGTCCAG-3′
AKHR-R	5′-AGTCGAACTGAGCCGCGAAA-3′
TUB-F	5′-CACCGGCTCTGGGTTCACTT-3′
TUB-R	5′-GAGATGACCGGTGCGTAGGTG-3′
RPS11-F	5′-TCGCTCATCACGCAACAACA-3′
RPS11-R	5′-ACGAGACAGGCGTTGTGTCC-3′
*For qRT-PCR*	
qAKH-F	5′-CCCTTCTGATGGCAGTCCTTTG-3′
qAKH-R	5′-ATGGATGCCTTGCAGCCTTCT-3′
qAKHR-F	5′-GCAGTGCTGAAGCCGATGAA-3′
qAKHR-R	5′-GGATGAGCCTGCACCCTGAA-3′
qTUB-F	5′-ACTCGTTCGGAGGAGGCACC-3′
qTUB-R	5′-GTTCCAGGGTGGTGTGGGTGGT-3′
qRPS11-F	5′-CCGATCGTGTGGCGTTGAAGGG-3′
qRPS11-R	5′-ATGGCCGACATTCTTCCAGGTCC-3′
*For dsRNA synthesis*	
AKHR-Fi	5′-ggatcctaatacgactcactatagggTTCACCGTCCTTTCCATCCTC-3′
AKHR-Ri	5′-ggatcctaatacgactcactatagggGAATCCTAAACTGGACCGACG-3′
GFP-Fi	5′-ggatcctaatacgactcactatagggAAGGGCGAGGAGCTGTTCACCG-3′
GFP-Ri	5′-ggatcctaatacgactcactatagggCAGCAGGACCATGTGATCGCGC-3′

*F, forward primer; R, reverse primer. Lowercase letters indicate the T7 promoter sequences*.

### RNA Interference and Bioassay

The RNA interference experiment was performed as described previously (Liu et al., 2010). Briefly, double-stranded RNA (dsRNA) was synthesized using T7 RiboMAX^TM^ Express RNAi System (Promega) with the specific primers linked by the T7 promoter sequence at the 5′ end. The integrity of dsRNA was confirmed by 1.5% agarose gel electrophoresis and dsRNA concentration was determined by a spectrophotometer NanoDrop 2000C (Thermo Fisher Scientific). The conjunctive between prothorax and mesothorax was selected as the dsRNA injection site. Newly emerged females (within 24 h) were anesthetized by carbon dioxide and injected with 23 nL dsRNA (about 100 ng) against the *NlAKHR* sequence or a control dsRNA designed against a green fluorescent protein (GFP) gene (ACY56286) using a Nanoject II microinjection device (Drummond Scientific, Broomall, PA, United States). The knockdown efficiency of *NlAKHR* was measured at 24 and 48 h after dsRNA injection using RT-PCR and qRT-PCR as describe above. Females injected with dsRNA were starved and the number of dead females was counted every 8 h. One hundred individuals were used in each set of repetition and three independent biological replicates were performed in RNAi experiments.

### *Nl*AKH Treatment

*Nl*AKH (pQVNFSPNW-NH_2_) was chemically synthesized (GenScript Biotech Inc., Nanjing, China) and dissolved in dimethyl sulfoxide (DMSO). In the present study, 20 pmol of *Nl*AKH was injected twice daily into newly emerged females (within 24 h) using a Nanoject II microinjection device (Drummond Scientific). Females injected with the same volume of DMSO were used as experimental controls. The effects of *Nl*AKH injection on the mobilization of lipid reserves were measured on the third day after treatment. Hemolymph and fat bodies were collected from thirty females in each replicate and three independent biological replicates were performed.

### Lipid Determination

Lipids were extracted as described previously, with moderate modification (Lorenz, 2003; Konuma et al., 2012). Briefly, hemolymph or fat bodies dissected from thirty females were mixed with 100 μL of 75% methanol, containing 10 mg of sodium sulfate and homogenized in a 300 μL mixture of chloroform/methanol (1:1), and then centrifuged at 12000 ×*g* at 4°C for 10 min. The supernatant was removed into a new tube and mixed with 150 μL of chloroform and 250 μL of 1 M NaCl, and the solvent was evaporated using a vacuum with centrifugation. The lipids from the organic layer were used for lipid quantification using a standard sulfo-phospho-vanillin method (Van, 1985; Xu et al., 2013).

Extracted lipids in chloroform/methanol (2 μL) mixed with 1 mL of sulfuric acid were heated at 100°C for 10 min and then cooled to room temperature, followed by adding 1 mL of vanillin reagents (0.2% vanillin in 67% ortho-phosphoric acid). Samples were measured at 540 nm using a NanoDrop 2000C spectrophotometer (Thermo Fisher Scientific), and lipid content was calculated against a lipid standard (cholesterol). Lipids derived from hemolymph and fat bodies were quantified as 1, 2-diacylglycerol (DAG) and TAG, respectively.

### Nile-Red Staining

Fat bodies were dissected in the precooled PBS buffer (pH 7.4) and the adherent tissues were carefully removed with forceps as thoroughly as possible under a stereomicroscope (SMZ745, Nikon). The dissected fat bodies were fixed with 4% paraformaldehyde on a glass slide for 2 h at room temperature and then washed with PBS for three times (3 min × 5 min). For lipid staining, fat bodies were submerged in Nile red solution [1 μL of Nile red (1 mg/mL) in 100 μL of PBS] and visualized using a Ti-S inverted fluorescence microscope (Nikon) within 2 h.

### Statistical Analysis

Results were presented as means ± SE (standard error) based on at least three independent biological replications. Differences between two groups were analyzed by Student’s *t*-test. One-way ANOVA followed by Duncan’s multiple comparison was used for the comparison among more than two different conditions. *P*-values less than 0.05 (^∗^) or 0.01 (^∗∗^) were considered to be statistically significant. Graphical representations and all statistical analyses were performed using GraphPad Prism 7.0 software (GraphPad Software, San Diego, CA, United States).

## Results

### Gene Identification and Phylogenetic Analysis

**FIGURE 1:**
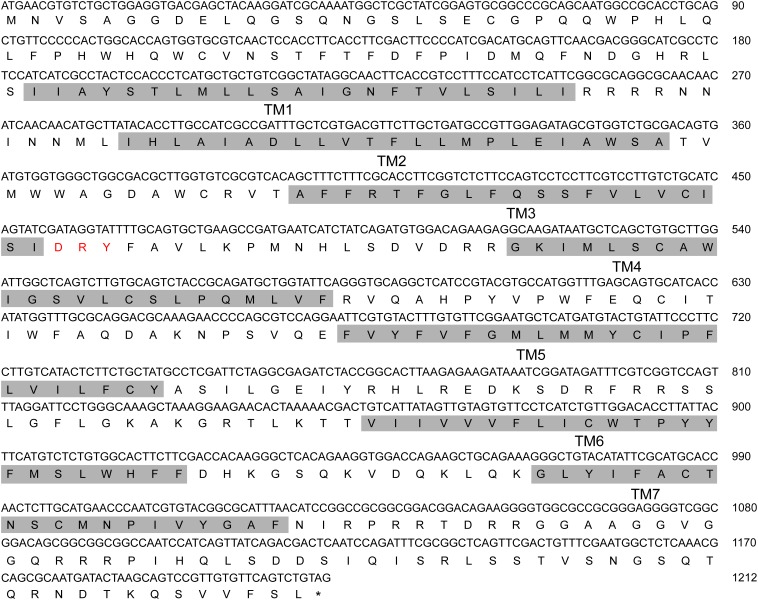
Nucleotide and its deduced amino acid sequence of *Nilaparvata lugens* adipokinetic hormone receptor (*Nl*AKHR). The numbering for each sequence is marked on the right. Seven predicted transmembrane (TM) regions 1–7 are shaded in gray. Red: amino acid residues predicted for AKHR signaling transduction and G-protein coupling. The stop codon is labeled by an asterisk.

**FIGURE 2:**
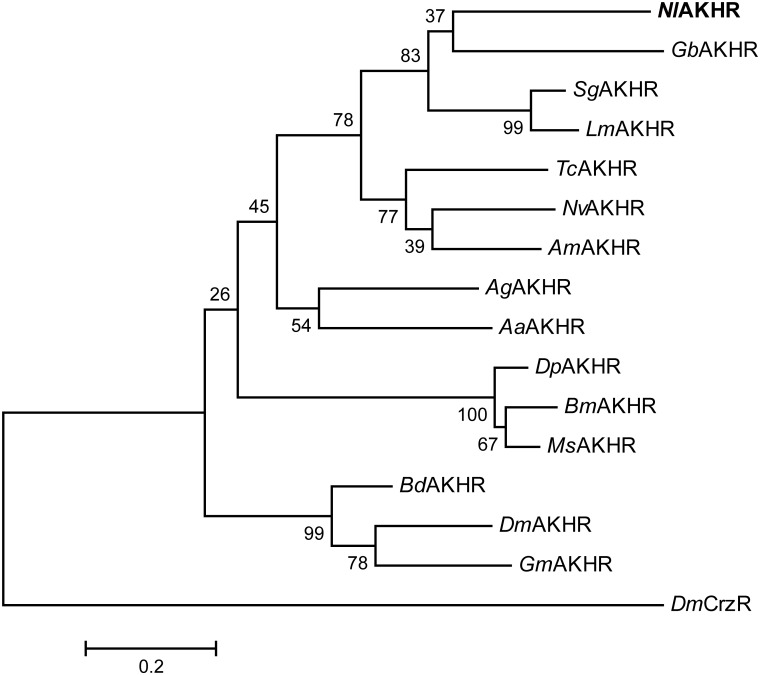
Phylogenetic tree of *Nl*AKHR and other insect AKHRs. The amino acid sequences of AKHR from *N. lugens* (*Nl*AKHR, MH238458), *Aedes aegypti* (*Aa*AKHR, CAY77164), *Anopheles gambiae* (*Ag*AKHR, ABD60146), *Apis mellifera* (*Am*AKHR, NP_001035354), *Bactrocera dorsalis* (*Bd*AKHR, AQX83416), *Bombyx mori* (*Bm*AKHR, NP_001037049), *Drosophila melanogaster* (*Dm*AKHR, NP_995639), *Danaus plexippus* (*Dp*AKHR, OWR46881), *Glossina morsitans* (*Gm*AKHR, AEH25943), *Gryllus bimaculatus* (*Gb*AKHR, ADZ17179), *Locusta migratoria* (*Lm*AKHR, ANW09575), *Manduca sexta* (*Ms*AKHR, AEH25943), *Nasonia vitripennis* (*Nv*AKHR, NP_001161243), *Schistocerca gregaria* (*Sg*AKHR, AVG47955), and *Tribolium castaneum* (*Tc*AKHR, NP_001076809) were aligned using the ClustalW program. The tree was constructed by MEGA 6 using the Maximum Likelihood (ML) method with 1000 bootstrap replicates. *D. melanogaster* Corazonin receptor (*Dm*CrzR, AF373862) was used as external group. The scale bar represents 0.2 amino acid substitutions per site.

*NlAKH* (AB817235) was identified in a previous study (Tanaka et al., 2014) and *NlAKHR* (MH238458) was cloned in this work. The full-length sequence of *NlAKHR* is 1610 bp, including a 1212 bp open reading frame (ORF) that encodes a protein consisting of 403 amino acid residues. No signal peptide cleavage was found in *Nl*AKHR. Seven transmembrane domains (TM) were identified in *Nl*AKHR, which indicates that this protein is a member of the GPCR superfamily (Figure 1). Alignment of *Nl*AKHR with other insect AKHRs showed that the transmembrane domain regions possessed particularly high conservation. The conserved sequence of DRY (positions 153–155), which was suggested to participate in AKHR signaling transduction and G-protein coupling, was identified (Wess, 1997). Global alignment showed that *Nl*AKHR possessed a highly conserved structure and sequence homologies with other known insect AKHRs, and the deduced amino acid sequence of *Nl*AKHR was approximately 60% identical to those described previously. Phylogenetic analysis showed that *Nl*AKHR and AKHRs from other insect species clustered in a group, and *Nl*AKHR is most closely related to *G. bimaculatus* homologs (*Gb*AKHR) (Figure 2).

### Spatial and Developmental Expression Profiles of *NlAKHR* and *NlAKH*

**FIGURE 3:**
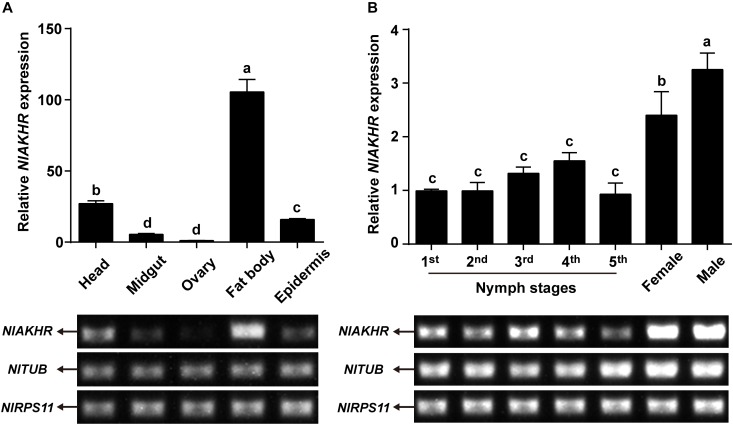
Expression analyses of *NlAKHR* in different tissues and developmental stages. **(A)** qRT-PCR and RT-PCR analyses of *NlAKHR* expression levels in different tissues from 3-day-old adult females. **(B)** qRT-PCR and RT-PCR analyses of *NlAKHR* expression levels in fat bodies from the first instar nymph to adults. Results are represented as means ± SE of three independent samples, and samples are normalized to *TUB* and *RPS11* expression levels. Different lowercase letters represent significant difference of *NlAKHR* levels among various tissues and developmental stages determined by one-way ANOVA followed by Duncan’s multiple comparison test (*P* < 0.05).

**FIGURE 4:**
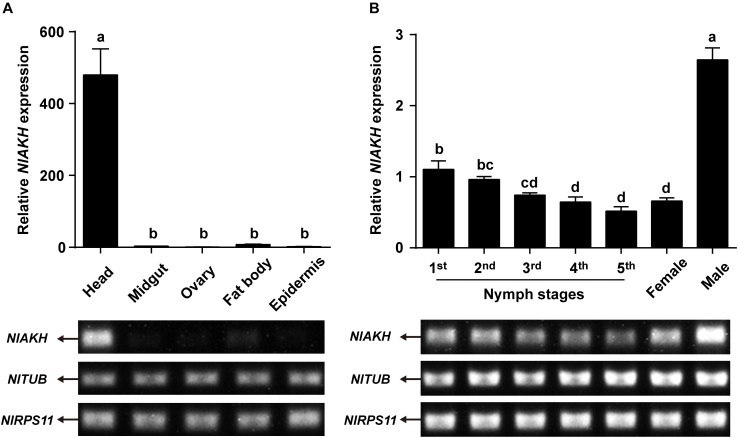
Expression analyses of *NlAKH* in different tissues and developmental stages. **(A)** qRT-PCR and RT-PCR analyses of *NlAKH* expression levels in different tissues from 3-day-old adult females. **(B)** qRT-PCR and RT-PCR analyses of *NlAKH* expression levels in heads from the first instar nymph to adults. *TUB* and *RPS11* were used as normalization controls. Results are displayed as means ± SE of three independent replicates. Different lowercase letters represent significant difference of *NlAKH* levels among various tissues and developmental stages determined by one-way ANOVA followed by Duncan’s multiple comparison test (*P* < 0.05).

Expression patterns of *NlAKHR* and *NlAKH* in different tissues and developmental stages were determined by RT-PCR and qRT-PCR. For the tissue-specific expression patterns, different tissues were dissected from 3-day-old females. The highest transcript level of *NlAKHR* was detected in fat body, followed by head and epidermis, with lower levels in the midgut and ovary (Figure 3A). It should be noted that part of the *NlAKHR* expression found in the head and epidermis of female *N. lugens* is attributable to the existence of fat body cells in these two organs. Developmental expression profile results showed that *NlAKHR* was highly expressed in adult males and females, with relatively lower levels in nymphal stages (Figure 3B). In contrast, *NlAKH* was exclusively expressed in the head, but not in the other tissues (Figure 4A). The highest expression of *NlAKH* was observed in adult males, with the lower levels in other nymphal stages and females (Figure 4B).

### Effects of *NlAKHR* Knockdown and *Nl*AKH Injection on Female Starvation Resistance, Lipid Content and Body Weight

**FIGURE 5:**
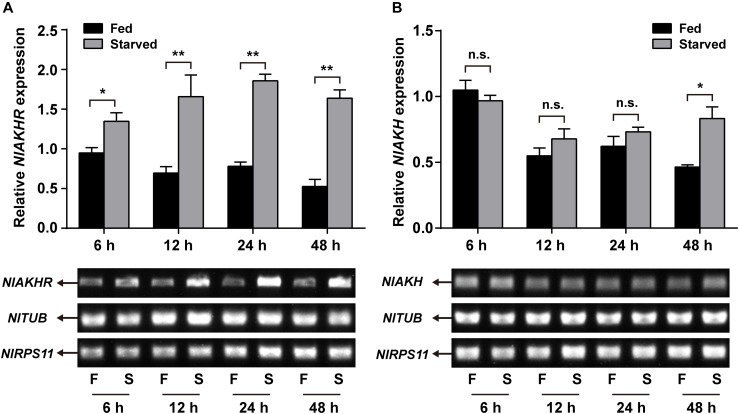
Effects of starvation on the expression of *NlAKHR* **(A)** and *NlAKH* **(B)**. Newly emerged females (within 24 h) were fed (F) or starved (S) for different hours. Differences between mRNA levels were determined by qRT-PCR and RT-PCR. *TUB* and *RPS11* were used as internal reference controls. Results are displayed as means ± SE of three independent replicates and asterisks indicate significant differences between the fed and starved groups at the same time point (^∗^*P* < 0.05 and ^∗∗^*P* < 0.01) by Student’s *t*-test. n.s. represents no significant difference (*P* > 0.05).

**FIGURE 6:**
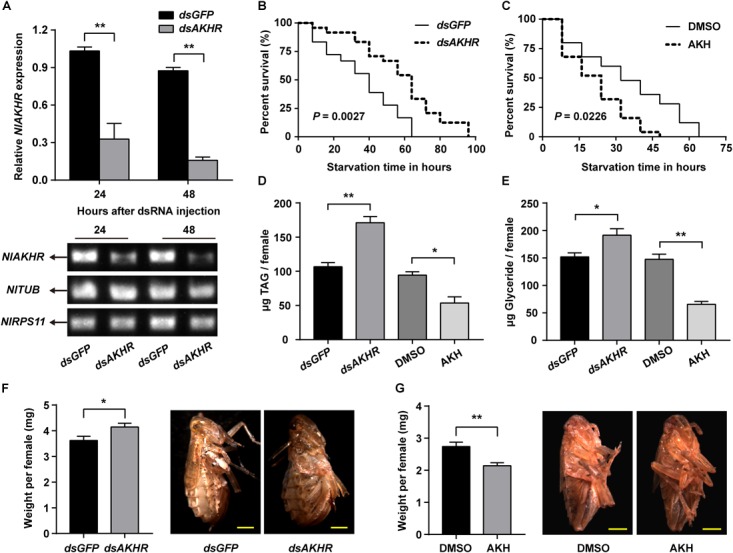
Effects of *NlAKHR* knockdown and AKH injection on the starvation resistance, whole body lipids and body weight. For RNAi, newly emerged females (within 24 h) were injected with 100 ng of dsRNA against *NlAKHR* (*dsAKHR*) or with a control dsRNA (*dsGFP*). For AKH treatment, newly emerged females were injected with 20 pmol of AKH or DMSO (control). **(A)** Fat bodies were dissected at 24 and 48 h after dsRNA injection. Differences between *NlAKHR* expression levels were determined by qRT-PCR and RT-PCR. *TUB* and *RPS11* were used as internal reference controls. Survival rates for *dsAKHR*-injected females **(B)** and AKH-treated females **(C)** under starvation condition were analyzed with Kaplan-Meier plot with a log rank test. TAG **(D)** and glyceride **(E)** contents in the whole body of *N. lugens* were determined at 48 h after injection. **(F)** Body weight of females was measured on the sixth day after dsRNA injection (*n* = 23–28). **(G)** Body weight of females was measured on the third day after AKH treatment (*n* = 12). Results are represented as means ± SE of three independent replicates and asterisks indicate significant differences (^∗^*P* < 0.05 and ^∗∗^*P* < 0.01) by Student’s *t*-test. Scale bar, 0.5 mm.

Compared to normally fed females, the gene expression levels of *NlAKHR* increased significantly by 1.4-, 2.4-, 2.4-, and 3.1-fold at 6 (*P* = 0.035), 12 (*P* = 0.028), 24 (*P* = 0.001) and 48 h (*P* < 0.001) after starvation, respectively (Figure 5A). The expression levels of *NlAKH* were elevated significantly, by 1.8-fold at 48 h after starvation (*P* = 0.015) (Figure 5B). To further confirm the roles of the AKH signaling system in lipid metabolism and starvation resistance in *N. lugens*, the dsRNA-mediated knockdown of *NlAKHR* was performed. The *dsAKHR* treatment of females resulted in a reduction of *NlAKHR* transcripts by 68.1% (*P* = 0.005) and 81.9% (*P* < 0.001) compared to *dsGFP*-injected controls at 24 and 48 h after dsRNA injection, respectively (Figure 6A). Females under starvation conditions after *NlAKHR* knockdown lived 24 h longer than the *dsGFP*-injected controls (*P* = 0.0027) (Figure 6B). AKH injection significantly reduced the median lifespan of females by 40% after starvation compared to DMSO-injected controls (*P* = 0.0226) (Figure 6C). Knockdown of *NlAKHR* led to an excessive accumulation of TAG (1.6-fold) (*P* = 0.004) and glyceride (1.3-fold) (*P* = 0.047) compared to the *dsGFP*-injected controls at 48 h after dsRNA injection. Conversely, AKH injection significantly depleted the TAG and glyceride contents of starved females by 43.2% (*P* = 0.016) and 55.6% (*P* = 0.002), respectively (Figures 6D,E). Females lacking AKHR function showed signs of obesity, accumulating 12.7% more body weight compared to the *dsGFP*-injected controls (*P* = 0.036) (Figure 6F). On the contrary, AKH injection, which accelerated lipid mobilization, resulted in a 21.9% reduction of body weight and produced slim females (*P* = 0.001) (Figure 6G).

### Effects of *NlAKHR* Knockdown and *Nl*AKH Injection on Lipid Mobilization

**FIGURE 7:**
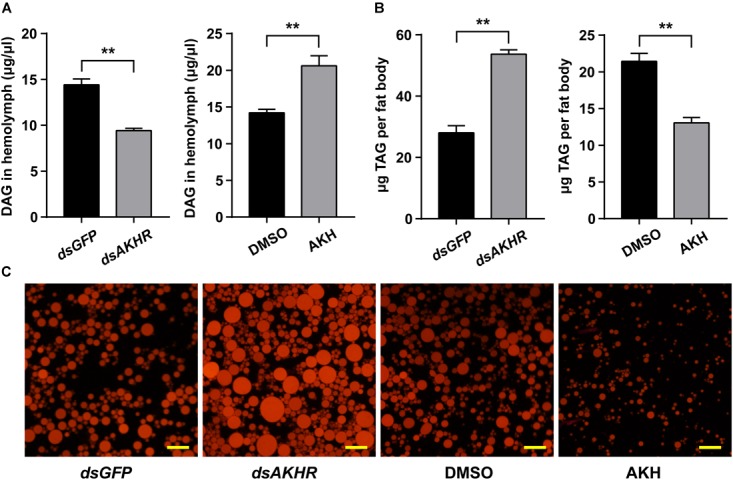
Effects of *NlAKHR* knockdown and AKH injection on the TAG mobilization. For RNAi, newly emerged females (within 24 h) were injected with 100 ng of dsRNA against *NlAKHR* (*dsAKHR*) or with a control dsRNA (*dsGFP*). For AKH treatment, 20 pmol of *Nl*AKH was injected twice daily into newly emerged females, and females injected with the same volume of DMSO were used as experimental controls. The amounts of DAG in the hemolymph **(A)** and TAG in the fat body **(B)** were determined on the third day after treatment. Results are represented as means ± SE of three independent replicates and asterisks indicate significant differences (^∗^*P* < 0.05 and ^∗∗^*P* < 0.01) by Student’s *t*-test. DAG, diacylglycerol; TAG, triacylglycerol. **(C)** Representative images from fat bodies after Nile red staining. Scale bar, 20 μm.

Knockdown of *NlAKHR* resulted in reduced levels of DAG (34.6% decrease) (*P* < 0.001) in the hemolymph compared with the *dsGFP*-injected controls. Conversely, the increased lipid levels in the hemolymph were confirmed by the results that AKH injection led to a critical accumulation of DAG (1.45-fold increase) in the hemolymph (*P* = 0.002) (Figure 7A). TAG levels in the fat body of *NlAKHR*-silenced females significantly increased compared to that of *dsGFP*-treated controls (1.9-fold increase) (*P* < 0.001), whereas AKH injection resulted in a 39.1% reduction of TAG content in the fat body (*P* < 0.001) (Figure 7B). To measure the stored lipid reserves in the fat body, Nile-red staining was performed to visualize the lipid droplets. As shown in Figure 7C, the size and number of visualized lipid droplets critically increased after *NlAKHR* knockdown, whereas AKH injection resulted in a critical reduction of lipid storage droplets in the fat body compared with the DMSO-treated controls.

## Discussion

Two AKHRs, which only differ at their C-terminus by containing phosphorylation sites for GPCR internalization, were identified in *Aedes aegypti* (Kaufmann et al., 2009) and *G. morsitans* (Attardo et al., 2012). However, in the present study, only a single copy gene that exhibits a high degree of homology to other AKHRs, was identified from *N. lugens*. Several lines of evidence support the idea that the putative *Nl*AKHR reported here is indeed an insect AKHR. Firstly, seven transmembrane domains that are involved in GPCR ligand binding and receptor activation are functionally conserved in *Nl*AKHR. In addition, it contains specific amino acid motifs typical for the GPCR family (Gonzalez et al., 2012). Secondly, the isolated *Nl*AKHR was highly analogous to other receptors that have been functionally characterized as AKHR from various insect species. Furthermore, *NlAKHR* knockdown resulted in decreased levels of circulating DAG in the hemolymph and an accumulation of TAG in the fat body. Our results clearly demonstrate that AKHR is critical for the maintenance of energy homeostasis, possibly due to the structural and functional conservation of the AKH signaling system in the regulation of lipolysis.

Adipokinetic hormone neuropeptides are primarily synthesized in the corpora cardiaca and are responsible for lipid mobilization during energy-demanding processes in a wide diversity of insect species (Auerswald et al., 2005; Auerswald and Gäde, 2006). In *D. melanogaster*, only a single AKH gene was identified (Gäde, 2010), while two AKH precursors that possess a similar structure typical of the AKH family were identified from the tsetse fly *G. morsitans* (Attardo et al., 2012) and the mosquito *A. aegypti* (Kaufmann et al., 2009). Both tsetse AKH peptides are the cognate ligands of *Gm*AKHR, indicating these two *AKH* genes derived from recent gene duplication (Caers et al., 2016). *Nl*AKH is exclusively expressed in the head of *N. lugens*, as has been demonstrated to be present in several insect species (Siegert, 1999; Kaufmann and Brown, 2006; Kaufmann et al., 2009). We also observed the highest expression levels of *NlAKH* in adult males of *N. lugens*, which indicates an interesting role for AKH in the energy mobilization associated with flight and, in particular, the reproduction of males. These high expression levels suggest a conserved function of the AKH signaling system in the regulation of lipid mobilization and energy homeostasis. *NlAKHR* was mostly expressed in the fat body of adult females, as also observed in *A. aegypti* (Kaufmann et al., 2009), *Anopheles gambiae* (Kaufmann and Brown, 2006), *D. melanogaster* (Staubli et al., 2002), and *Manduca sexta* (Ziegler et al., 2011). This corresponds with the main function of AKH on lipid and carbohydrate mobilization from the fat body under conditions of high energy demand (Marco et al., 2013). In insects, the fat body is the main organ for lipid storage and energy utilization (Arrese and Soulages, 2010). The highest expression level in the fat body also suggests a conserved role of AKHR in the regulation of energy mobilization in *N. lugens*.

Many studies have demonstrated that the AKH signaling system plays an important role in lipid mobilization. Nutritional status, either feeding or starvation, can significantly affect the expression of AKH signaling system components. For example, *AKHR* transcript levels decreased after protein meals and it is likely that TAG synthesis may be stimulated and lipolysis may be inhibited in the blood-fed mosquito *A. gambiae* (Kaufmann and Brown, 2006) and liver-fed flesh fly *S. crassipalpis* (Bil et al., 2016). It also corresponds well with the decrease of AKH neuropeptides observed in the liver fed *S. crassipalpis* (Bil et al., 2014). Here, we found that starvation induces the expression of *AKH* and its receptor *AKHR*, indicating that the AKH signaling system is involved in the regulation of starvation resistance. It seems likely that the higher abundance of AKH and AKHR stimulates the use of stored lipid reserves and meets energy demands when food is unavailable. In *D. melanogaster*, AKHR mutant flies possessed high levels of lipids at the time of death, and this is likely due to the inability of flies to mobilize stored lipids under starvation conditions (Grönke et al., 2007). Our results showed that knockdown of *NlAKHR* resulted in extended survival during starvation and accumulated lipid reserves, as previously observed in *D. melanogaster* (Grönke et al., 2007) and *G. bimaculatus* (Konuma et al., 2012). Conversely, the rate of lipolysis under starvation conditions accelerated and starvation resistance decreased after AKH exposure. Since the lipid reserves deposited in the fat body are the main energy resources under food-deprived conditions, it is likely that AKH signaling system-mediated lipolysis is essential for starvation resistance. *NlAKHR* knockdown females appear incapable of mobilizing lipid reserves, resulting in an obese phenotype. Similar results have been obtained in the cricket *G. bimaculatus*, wherein knockdown of *AKHR* resulted in an increase of TAG in the fat body and a decrease of DAG in the hemolymph (Konuma et al., 2012). *AKHR* mutants of *D. melanogaster* also accumulated high levels of TAG in the fat body (Grönke et al., 2007; Bharucha et al., 2008). These results support the idea that TAG mobilization is closely related to AKHR expression, and the AKH signaling system is critical for maintaining energy homeostasis. Here, we demonstrate that knockdown of *NlAKHR* increased the TAG levels in the fat body and decreased circulating DAG contents in the hemolymph. Conversely, *Nl*AKH-treated females are slim with a severe reduction in stored lipid levels. This phenotype is a probable result of reduced fat body lipid reserves during starvation as AKH accelerates lipid mobilization. The inability to accumulate lipid reserves after AKH exposure was demonstrated in the locust *Schistocerca gregaria*, and the mosquito *A. aegypti* (Ziegler, 1997; Gokuldas et al., 2010). In addition, the exposure of *Nl*AKH also affected the distribution of lipids, decreasing TAG levels in the fat body while increasing circulating DAG levels in the hemolymph. Based on these results, we speculate here that lipid mobilization in the fat body is tightly regulated by the AKH signaling system, which is essential in adjusting body lipid homeostasis and ensuring energy supplementation during starvation in *N. lugens*.

Two lipolytic systems, including the AKH/AKHR signaling system and brummer lipase system, are conserved in *N. lugens*, as has been shown in *D. melanogaster* (Grönke et al., 2005, 2007) and *G. morsitans* (Attardo et al., 2012). These two pathways are somewhat functionally redundant in regard to lipid mobilization; that is, when one pathway is suppressed the other may account for partial compensation. Our previous studies have demonstrated that brummer lipase plays an important role in maintaining hemolymph lipid levels during the starvation period (Zhou et al., 2018a). Given the scale of lipid mobilization required for survival during periods of nutritional stress, it appears that both the AKH/AKHR and brummer pathways are critical for lipid mobilization and starvation resistance (Grönke et al., 2005, 2007). The combination of these two lipolytic systems in *N. lugens* provides the flexibility to resist various kinds of stress by utilizing the strengths of one system to compensate for the weakness of the other. The molecular mechanisms that govern the cross-talk between these two lipolytic pathways in the regulation of lipid homeostasis and starvation resistance are the focus of research in our laboratory.

## Author Contributions

KL and QZ designed the research and wrote the paper. XZ, XC, YL, WL, and YC performed the experiments and analyzed the data. JZ and KY revised the manuscript. All authors listed have approved the manuscript for publication.

## Conflict of Interest Statement

The authors declare that the research was conducted in the absence of any commercial or financial relationships that could be construed as a potential conflict of interest.
