# Supplementary material for: Adipokinetic Hormone Receptor Mediates Lipid Mobilization to Regulate Starvation Resistance in the Brown Planthopper, Nilaparvata lugens
Source: Front Physiol. 2018 Nov 29;9:1730. doi: 10.3389/fphys.2018.01730 (PMC6281999; doi:10.3389/fphys.2018.01730)
Supplement: Supplementary file 1 [file Table_1.DOCX]

**Supplementary Table 1**. List of AKHR from different insect species.

| Order | Insect species | References |
| --- | --- | --- |
| Blattoptera | *Blattella Americana* | Huang et al., 2011 |
|  | *Periplaneta americana* | Hansen et al., 2006 |
|  |  | Wicher et al., 2006 |
| Coleoptera | *Tribolium castaneum* | Li et al., 2008 |
| Diptera | *Aedes aegypti* | Kaufmann et al., 2009 |
|  | *Anopheles gambiae* | Kaufmann and Brown, 2006 |
|  | *Bactrocera dorsalis* | Hou et al., 2017 |
|  | *Glossina morsitans* | Attardo et al., 2012 |
|  | *Sarcophaga crassipalpis* | Bil et al., 2016 |
| Hemiptera | *Acyrthosiphon pisum* | Li et al., 2013 |
|  | *Pseudoregma bambucicola* | Jedličková et al., 2015 |
|  | *Rhodnius prolixus* | Alves-Bezerra et al., 2016 |
| Hymenoptera | *Bombus terrestris* | Jedlička et al., 2016 |
|  | *Nasonia vitripennis* | Hansen et al., 2010 |
| Lepidoptera | *Manduca sexta* | Ziegler et al., 2011 |
| Orthoptera | *Gryllus bimaculatus* | Konuma et al., 2012 |

**REFERENCES**

Alves-Bezerra, M., Paula, I.F.D., Medina, J.M., Silva-Oliveira, G., Medeiros, J.S., Gäde, G., et al. (2016). Adipokinetic hormone receptor gene identification and its role in triacylglycerol metabolism in the blood-sucking insect *Rhodnius prolixus*. *Insect Biochem. Mol. Biol.* 69, 51-60. doi: 10.1016/j.ibmb.2015.06.013

Attardo, G.M., Benoit, J.B., Michalkova, V., Yang, G., Roller, L., Bohova, J., et al. (2012). Analysis of lipolysis underlying lactation in the tsetse fly, *Glossina morsitans*. *Insect Biochem. Mol. Biol.* 42, 360-370. doi:10.1016/j.ibmb.2012.01.007

Bil, M., Timmermans, I., Verlinden, H., and Huybrechts, R. (2016). Characterization of the adipokinetic hormone receptor of the anautogenous flesh fly, *Sarcophaga crassipalpis*. *J. Insect Physiol*. 89, 52-59. doi: 10.1016/j.jinsphys.2016.04.001

Hansen, K.K., Hauser, F., Cazzamali, G., Williamson, M., and Grimmelikhuijzen, C.J. (2006). Cloning and characterization of the adipokinetic hormone receptor from the cockroach *Periplaneta americana*. *Biochem. Biophys. Res. Commun*. 343, 638-643. doi: 10.1016/j.bbrc.2006.03.012

Hansen, K.K., Stafflinger, E., Schneider, M., Hauser, F., Cazzamali, G., Williamson, M., et al. (2010). Discovery of a novel insect neuropeptide signaling system closely related to the insect adipokinetic hormone and corazonin hormonal systems. *J. Biol. Chem*. 285, 10736-10747. doi: 10.1074/jbc.M109.045369

Hou, Q.L., Chen, E.H., Jiang, H.B., Wei, D.D., Gui, S.H., Wang, J.J., et al. (2017). Adipokinetic hormone receptor gene identification and its role in triacylglycerol mobilization and sexual behavior in the oriental fruit fly (*Bactrocera dorsalis*). *Insect Biochem. Mol. Biol.* 90, 1-13. doi: 10.1016/j.ibmb.2017.09.006

Huang, J.H., Xavier, B., and How-Jing, L. (2011). Functional characterization of hypertrehalosemic hormone receptor in relation to hemolymph trehalose and to oxidative stress in the cockroach *Blattella germanica*. *Front. Endocrinol*. 2, 114. doi: 10.3389/fendo.2011.00114

Jedlička, P., Ernst, U.R., Votavová, A., Hanus, R., and Valterová, I. (2016). Gene expression dynamics in major endocrine regulatory pathways along the transition from solitary to social life in a bumblebee, *Bombus terrestris*. *Front. Physiol*. 7, 574. doi: 10.3389/fphys.2016.00574

Jedličková, V., Jedlička, P., and Lee, H.J. (2015). Characterization and expression analysis of adipokinetic hormone and its receptor in eusocial aphid *Pseudoregma bambucicola*. *Gen. Comp. Endocrinol*. 223, 38-46. doi: 10.1016/j.ygcen.2015.09.032

Kaufmann, C., and Brown, M.R. (2006). Adipokinetic hormones in the African malaria mosquito, *Anopheles gambiae*: identification and expression of genes for two peptides and a putative receptor. *Insect Biochem. Mol. Biol.* 36, 466-481. doi: 10.1016/j.ibmb.2006.03.009

Kaufmann, C., Merzendorfer, H., and Gäde, G. (2009). The adipokinetic hormone system in Culicinae (Diptera: Culicidae): molecular identification and characterization of two adipokinetic hormone (AKH) precursors from *Aedes aegypti* and *Culex pipiens* and two putative AKH receptor variants from *A. aegypti*. *Insect Biochem. Mol. Biol.* 39, 770-781. doi: 10.1016/j.ibmb.2009.09.002

Konuma, T., Morooka, N., Nagasawa, H., and Nagata, S. (2012). Knockdown of the adipokinetic hormone receptor increases feeding frequency in the two-spotted cricket *Gryllus bimaculatus*. *Endocrinology* 153, 3111-3122. doi: 10.1210/en.2011-1533

Li, B., Predel, R., Neupert, S., Hauser, F., Tanaka, Y., Cazzamali, G., et al. (2008). Genomics, transcriptomics, and peptidomics of neuropeptides and protein hormones in the red flour beetle *Tribolium castaneum*. *Genome Res*. 18, 113-122. doi: 10.1101/gr.6714008

Li, C., Yun, X., Hu, X., Zhang, Y., Sang, M., Liu, X., et al. (2013). Identification of G protein-coupled receptors in the pea aphid, *Acyrthosiphon pisum*. *Genomics* 102, 345-354. doi: 10.1016/j.ygeno.2013.06.003

Wicher, D., Agricola, H.J., Söhler, S., Gundel, M., Heinemann, S.H., Wollweber, L., et al. (2006). Differential receptor activation by cockroach adipokinetic hormones produces differential effects on ion currents, neuronal activity, and locomotion. J. Neurophysiol. 95, 2314-2325. doi: 10.1152/jn.01007.2005

Ziegler, R., Isoe, J., Moore, W., Riehle, M.A., and Wells, M.A. (2011). The putative AKH receptor of the tobacco hornworm, *Manduca sexta*, and its expression. *J. Insect Sci*. 11, 40. doi: 10.1673/031.011.0140
